# Impact of the COVID-19 Pandemic on the Long-Term Prognosis of Acute Myocardial Infarction in Japan

**DOI:** 10.7759/cureus.51905

**Published:** 2024-01-08

**Authors:** Hiroki Sato, Keisuke Yonezu, Shotaro Saito, Ichitaro Abe, Katsunori Tawara, Hidefumi Akioka, Tetsuji Shinohara, Yasushi Teshima, Kunio Yufu, Ryuzo Abe, Naohiko Takahashi

**Affiliations:** 1 Department of Cardiology and Clinical Examination, Faculty of Medicine, Oita University, Oita, JPN; 2 Advanced Trauma, Emergency and Critical Care Center, Oita University Hospital, Yufu, JPN; 3 Department of Cardiology and Clinical Examination, Faculty of Medicine, Oita University, Yufu, JPN; 4 Department of Emergency Medicine, Faculty of Medicine, Oita University, Oita, JPN

**Keywords:** survival analyses, all-cause death, major adverse cardiac effect (mace), acute myocardial infarcation, covid-19

## Abstract

Background

During the early phase of the coronavirus disease 2019 (COVID-19) pandemic, a global reduction in hospitalizations for acute myocardial infarction (AMI) was observed. Generally, patients experienced increased severity of AMI with delays in time from symptom onset to treatment during the pandemic. However, the impact of the COVID-19 pandemic on in-hospital mortality among patients with AMI remains unclear. This study aimed to compare the long-term prognosis of patients with AMI during the COVID-19 pandemic to that observed in the pre-pandemic period and to evaluate the influence of the COVID-19 pandemic on the prognosis of patients with AMI.

Methods

We reviewed the data of patients admitted to our hospital for AMI treatment between April 1, 2018, and March 31, 2021. The time from admission to major adverse cardiac events (MACE), as well as the time from admission to all-cause death, were examined between the pandemic period (April 1, 2020, to March 31, 2021) and the pre-pandemic period (April 1, 2018, to March 31, 2020).

Results

Eighty patients were included in the study, and those admitted during the pandemic exhibited a higher likelihood of advanced age, lower levels of LDL-cholesterol, and a reduced prevalence of hypertension. The 2.5-year MACE-free survival and overall survival rates between the patients during the pre-pandemic and pandemic periods were not significantly different.

Conclusion

The long-term prognosis of patients with AMI during the COVID-19 pandemic remains unclear. In this study, we reported that the 2.5-year MACE-free survival and overall survival rates of the patients with AMI admitted during the COVID-19 pandemic were not significantly different from those during the pre-pandemic period. The impact of the COVID-19 pandemic on the prognosis of patients with AMI appears to vary according to the study population.

## Introduction

A decline in hospitalizations for acute myocardial infarction (AMI) has been globally reported during the early phase of the coronavirus disease 2019 (COVID-19) outbreak [1,2]. This decrease is attributed, in part, to patients hesitating to seek medical care, possibly due to concerns about COVID-19 transmission and a commitment to adhere to social distancing measures [3]. This may be related to compromised access to healthcare systems and the diminished capacity of hospitals to provide routine medical care due to the pandemic [4].

The behavior changes of patients and the worsened access to healthcare services during the initial stages of the COVID-19 pandemic have been associated with treatment delays for AMI. The time from symptom onset to seeking medical services and the time from initial contact with medical services to treatment were extended during the early phase of the COVID-19 pandemic compared to the pre-pandemic period. Specifically, the time from symptom onset to first medical contact was significantly prolonged for patients with ST-elevation myocardial infarction (STEMI) [5]. In Korea, the door-to-balloon time for STEMI cases during the pandemic exceeded that observed before the pandemic [6]. The delayed time from the symptom onset to treatment initiation for AMI contributed to exacerbated severity in patients with AMI during the early phase of the pandemic [1].

The worsened severity of patients with AMI resulting from delayed treatment initiation during the pandemic may contribute to poor prognosis. The composite outcomes encompassing in-hospital death, cardiogenic shock, sustained ventricular tachycardia/ventricular fibrillation, and the use of mechanical support was significantly worse during the pandemic among patients with AMI [5]. This trend is further supported by evidence from a study in Italy, where in-hospital mortality and complication rates significantly increased during the early phase of the pandemic [7]. Some studies have suggested a compromised prognosis for patients with AMI amid the pandemic [8-10].

Nevertheless, the changes in mortality-related endpoints during the pandemic remain controversial. Some studies have suggested that in-hospital mortality for patients with AMI during the pandemic was not significantly different compared to the pre-pandemic period [11-14]. In Germany, the in-hospital mortality rates for STEMI and non-ST-elevated myocardial infarction (NSTEMI) did not differ before and during the pandemic [15]. Similarly, there was no significant difference in in-hospital mortality in Korea, where the door-to-balloon time for STEMI was longer during the pandemic [6].

In these studies, the mortality-related endpoints, such as in-hospital mortality and 30-day mortality, were used to evaluate the relatively short-term prognosis of patients with AMI during the pandemic [16-18]. However, the long-term prognosis of patients with AMI during the pandemic remains unclear.

This study aimed to evaluate the long-term prognosis of patients with AMI during the COVID-19 pandemic in comparison to that during the pre-pandemic period.

## Materials and methods

Data collection and management

This retrospective study utilized data from our hospital's electronic health records system. The study population comprised patients admitted to our institution for AMI treatment between April 1, 2018, and March 31, 2021. Inclusion criteria encompassed those undergoing initial admission for AMI during the study period. Admission records were collected based on the diagnosis of AMI, designated as either the main diagnosis, admission-precipitating diagnosis, or the most resource-consuming diagnosis within the Japanese Diagnosis Procedure Combination (DPC) database. The AMI diagnosis in the DPC database was defined according to the International Classification of Diseases-10 (ICD-10) codes under I21. The electronic health record system obtained the patient characteristics, laboratory results, history, coexisting conditions, treatment details, and prognostic information. We reviewed all electronic health records of patients diagnosed with AMI and excluded non-AMI cases from the study.

We divided the study period into two periods: the pre-pandemic period (April 1, 2018 to March 31, 2020) and the pandemic period (April 1, 2020 to March 31, 2021). This division aligns with the Japanese government's declaration of a state of emergency on April 7, 2020, in response to the COVID-19 pandemic [19].

Outcomes

The primary outcome was the time from the first admission for AMI treatment to the occurrence of major adverse cardiac events (MACE). This study defined MACE as a composite of total death, AMI, and stroke. Additionally, the secondary outcome focused on time from admission to all-cause death.

Statistical analysis

Continuous variables were presented as means with standard deviations or median with interquartile ranges, and their comparison was conducted using the Student's t-test or Wilcoxon rank-sum test, as deemed appropriate. Categorical variables were expressed as frequencies and percentages, and their comparisons were conducted using Fisher's exact test.

A Kaplan-Meier plot was constructed to demonstrate the difference in survival between patients admitted during the pre-pandemic and pandemic periods. The log-rank test was used to examine the relationship between the COVID-19 pandemic and the survival of patients with AMI. Univariate and multivariate Cox proportional hazards models were used. Statistical significance was set at P < 0.05. All statistical analyses were performed using SAS version 9.4 (SAS Institute, Inc., Cary, NC, USA).

Ethics

This study received approval from the Medical Ethics Committee of Oita University Hospital (no.2674). The study was conducted in accordance with the Declaration of Helsinki and the Ethical Guidelines for Medical and Health Research Involving Human Subjects, as provided by the Ministry of Health, Labour, and Welfare, Japan.

## Results

Patient characteristics

**Figure 1:**
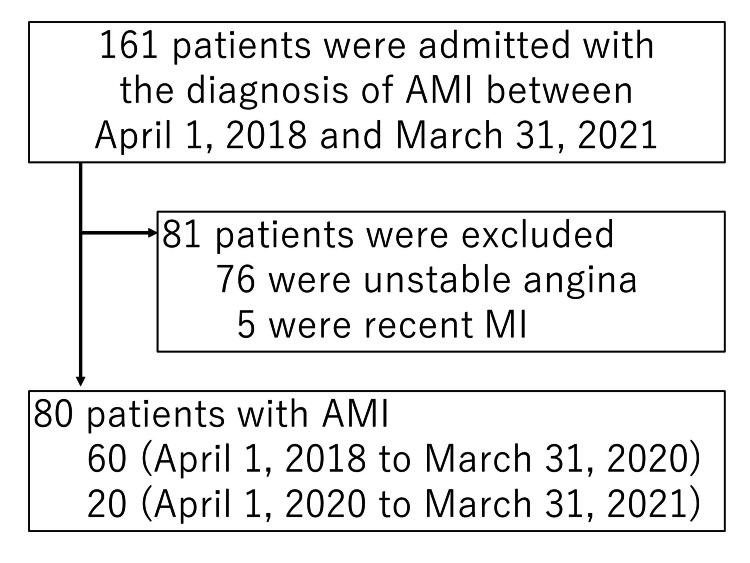
Flow diagram AMI: acute myocardial infarction

The study encompassed 80 hospitalizations due to AMI over the study period, with 60 patients admitted during the pre-pandemic period and 20 patients during the pandemic period (Figure 1). The baseline characteristics of the patient population are shown in Table 1. Patients admitted during the pandemic exhibited a higher likelihood of advanced age, lower levels of LDL-cholesterol, and a reduced prevalence of hypertension. Although the median NT-proBNP level in patients with AMI admitted during the pandemic period was higher than in the pre-pandemic period, this difference was not statistically significant. All patients underwent coronary angiography and percutaneous coronary intervention, except for four patients who opted for coronary artery bypass graft surgery. Notably, there were no COVID-19 cases in the study population.

**Table 1:** Baseline characteristics of patients with AMI Categorical data were presented as frequency (percentage). Continuous data were presented as mean±standard deviation or median (25 percentile, 75 percentile) as appropriate. STEMI, ST-elevation myocardial infarction; RCA, right coronary artery; LAD, left anterior descending artery; LCx, left circumflex artery; OHCA, out-of-hospital sudden cardiac arrest, VF, ventricular fibrillation; VSP, ventricular septal perforation; CABG, coronary artery bypass graft surgery; IABP. intra baloon pumping; VA-ECMO, veno-arterial extracorporeal membrane oxygenation; CK, creatine kinase, CK-MB, creatine kinase, eGFR, estimated glumonary renal function; ACE-I, angiotensin converting enzyme inhibitor; ARB, angiotensin receptor blocker; MRA, mineral recepter antagonist; SGLT-2, sodium-glucose cotransporter-2. * Statistically significant P < 0.05

Characteristic	Pre-pandemic (N = 60)	Pandemic (N = 20)	P value*
Male sex	47 (78.3%)	14 (70.0%)	0.173
Age, year-old	71.7 (11.6%)	77.9 (11.5%)	0.041
STEMI	45 (75.0%)	17 (85.0%)	0.538
Culprit vessel			
RCA	18 (30.0%)	7 (35.0%)	0.352
LAD	36 (60.0%)	9 (45.0%)	
LCx	6 (10.0%)	4 (20.0%)	
OHCA	3 (5.0%)	0 (0.0%)	0.569
VF	4 (6.7%)	1 (5.0%)	1.000
Mechanical complications			
VSP	2 (3.3%)	0 (0.0%)	1.000
cardiac tamponade	1 (1.7%)	1 (5.0%)	0.440
Intervention			
CABG	4 (6.7%)	0 (0.0%)	0.567
Mechanical circulatory support			
IABP	11 (18.3%)	3 (15.0%)	1.000
VA-ECMO	2 (3.3%)	2 (10.0%)	0.259
Impella	0 (0.0%)	2 (10.0%)	0.060
Laboratory data			
CK, U/L	189.0 (116.0, 665.0)	241.5 (162.5, 436.0)	0.288
CK-MB, ng/mL	15.9 (2.8, 63.2)	18.2 (6.3, 57.1)	0.509
CK peak, U/L	1486.0 (722.0, 2845.0)	1822.0 (595.0, 3521.5)	0.803
Troponin T, ng/mL	0.224 (0.040, 1.390)	0.142 (0.363, 0.528)	0.461
LDL-Cholesterol, mg/dL	121.7±40.2	100.4±40.6	0.044
HDL-Cholesterol, mg/dL	48.3±13.0	51.8±15.6	0.324
Creatinine, mg/dL	0.87 (0.72, 1.09)	0.89 (0.72, 1.31)	0.722
eGFR, ml/min/1.73m^2^	65.8±3.3	60.7±6.9	0.463
Hemoglobin, g/dL	13.6±2.0	13.0±2.0	0.193
NTproBNP, pg/mL	361.2 (121.4, 1452.0)	2147.0 (201.0, 7697.0)	0.121
Risk factors for cardiovascular diseases			
Current smoker	17 (28.3%)	4 (20.0%)	0.355
Former smoker	24 (40.0%)	6 (30.0%)	
Hypertension	38 (63.3%)	5 (25.0%)	0.004
Dyslipidemia	35 (58.3%)	10 (50.0%)	0.606
Diabetes Mellitus	14 (23.3%)	4 (20.0%)	1.000
Chronic kidney disease	5 (8.3%)	3 (15.0%)	0.405
Length of hospital stay, days	20.1±3.4	19.5±1.8	0.919
Medical treatment at the discharge			
ACE-I/ARB	48 (80.0%)	16 (80.0%)	1.000
Beta blocker	43 (71.7%)	14 (70.0%)	1.000
MRA	4 (6.7%)	6 (30.0%)	0.013
SGLT-2 inhibitor	5 (8.3%)	2 (10.0%)	1.000
statin	55 (91.7%)	18 (90.0%)	1.000

Survival of patients with AMI before and during the pandemic

**Figure 2:**
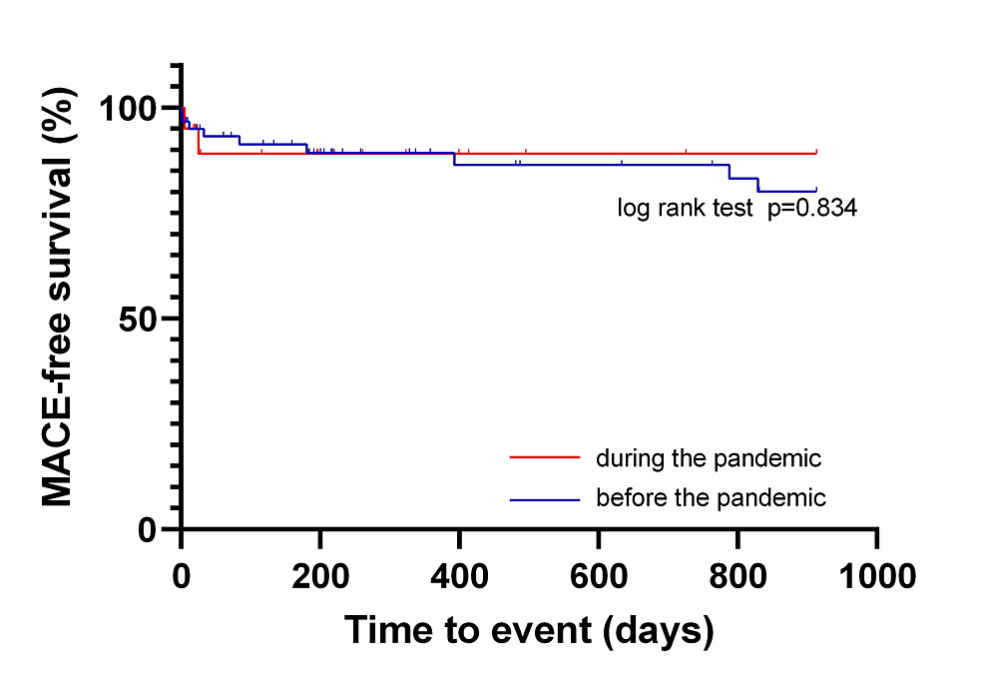
MACE-free survival rates of patients with AMI during the pre-pandemic and pandemic periods Statistically significant P < 0.05

**Figure 3:**
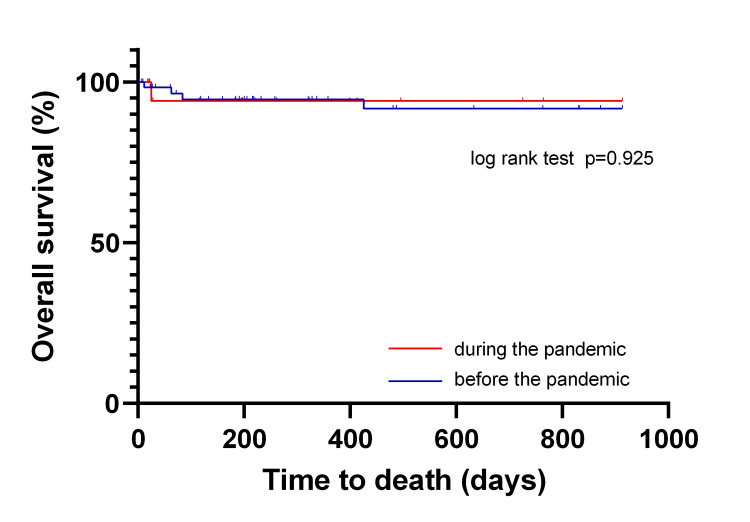
Overall survival rates of patients with AMI during the pre-pandemic and pandemic periods * Statistically significant P < 0.05

The 2.5-year MACE-free survival rates are illustrated in Figure 2. There was no significant difference in MACE-free survival between the patients in the pre-pandemic and pandemic periods. Additionally, the 2.5-year overall survival of patients with AMI before and during the pandemic exhibited no significant difference (Figure 3). Multiple Cox regression analysis demonstrated no significant difference in prognosis between the pre-pandemic and pandemic periods (Table 2).

**Table 2:** Cox proportional hazard regression analysis of patients with AMI during the pre-pandemic and the pandemic periods Hazard ratio has been represented as an estimated value with 95% confidential interval. HR, hazard ratio; CI, confidence interval. * Statistically significant P < 0.05

	Univariate analysis		Multivariate analysis	
	HR (95%CI)	p-value*	HR (95%CI)	p-value*
MACE-free survival				
COVID-19 pandemic	0.85 (0.18, 4.00)	0.834	0.52 (0.10, 2.59)	0.423
STEMI	-	-	0.71 (0.21, 2.46)	0.594
age, year	-	-	1.04 (0.98, 1.11)	0.233
LDL-cholesterol (/10mg/dL)	-	-	0.86 (0.72, 1.03)	0.105
Hypertension	-	-	0.83 (0.24, 2.83)	0.765
Overall survival				
COVID-19 pandemic	0.90 (0.10, 8.10)	0.925	0.55 (0.05, 5.75)	0.617
STEMI	-	-	0.58 (0.10, 3.46)	0.547
age, year	-	-	1.05 (0.96, 1.16)	0.290
LDL-cholesterol (/10mg/dL)	-	-	0.90 (0.69, 1.18)	0.439
Hypertension	-	-	1.10 (0.17, 7.24)	0.918

## Discussion

The 2.5-year MACE-free survival and overall survival rates of patients with AMI during the COVID-19 pandemic did not exhibit significant differences compared to those during the pre-pandemic period. The similar results from the multiple regression analysis confirmed this finding.

The short-term prognosis of patients with AMI amid the COVID-19 pandemic has been investigated worldwide. Some studies have suggested that in-hospital mortality during the pandemic was worse than during the pre-pandemic period [1]. Factors contributing to this trend include delayed time from symptom onset to treatment due to individual hesitation to visit hospitals for fear of contracting COVID-19, delays in transportation, and wait time for the results of COVID-19 screening tests [1,20]. In Japan, an increase in the proportion of mechanical complications in patients with STEMI with delayed reperfusion due to delayed hospital arrival during the pandemic has been reported [21]. Poor prognosis due to exacerbated severity of AMI is reasonable. However, findings from other studies revealed that the in-hospital mortality rate was not significantly different between the pre-pandemic and the pandemic periods [8-10].

These inconsistencies may be attributed to several factors. The presence of COVID-19 infection among patients with AMI appears to have an influence on the prognosis of patients during the pandemic. The COVID-19 infection causes myocardial injuries through pathways such as cytokine storm, endothelial damage, coagulopathy, and other factors [22]. Patients concurrently affected by both AMI and COVID-19 tend to experience poorer prognosis than those without COVID-19 infection [23]. Some studies have suggested that in-hospital mortality exhibited a worsened trend during the pandemic, although no significant difference was noted after excluding patients with AMI infected with COVID-19 [24,25]. Another study showed that even after adjusting for COVID-19, an analysis of patients with AMI still revealed a significantly worse prognosis during the pandemic [18,25]. Several studies, particularly those using large databases, did not conduct an analysis excluding AMI patients infected with COVID-19.

Additionally, numerous studies omitted the presentation of the count of patients infected with COVID-19 in the study population; however, COVID-19 infection itself could potentially influence the results in comparing outcomes between the pre-pandemic and pandemic periods. In this study, the impact of COVID-19 infection on AMI patients was not evaluated because there were no AMI patients infected with COVID-19 in the study population. This study evaluated the impact of changes in people's behavior, public health measures, worsened access to healthcare services, and other factors during the pandemic on the prognosis of patients with AMI.

Socioeconomic factors, medical resources, healthcare systems, and public health measures in each study population may also contribute to differences in the prognosis of patients with AMI patients during the pandemic. In the United States, for instance, education level and race/ethnicity have been identified to be correlated to the prognosis of patients with AMI [26,27]. Public health measures implemented in response to the COVID-19 pandemic, such as social distancing, school closures, lockdowns, and behavioral changes caused by these measures, are likely to influence the prognosis [28]. During the lockdown period, an increase in in-hospital mortality was observed [29].

The impact of the pandemic's scale on the prognosis of patients with AMI remains unclear. A comparative analysis of two prefectures, with different scales of pandemic and social campaigns, did not reveal a significant difference in in-hospital mortality [8].

In Japan, some reports have addressed the prognosis of patients with AMI. For instance, a single-center study in Tokyo reported that the 30-day cumulative mortality of STEMI patients during the COVID-19 pandemic was higher than that during the pre-pandemic period; however, the difference was not significant (13.7% of 117 patients vs. 6.6% of 281 patients, P = 0.074) [30]. In Mie Prefecture, Japan, the in-hospital mortality of patients with AMI during the early phase of the pandemic (April 2020 to September 2020) was not significantly different from that during the pre-pandemic period [20].

Results from a meta-analysis indicated a significant increase in in-hospital mortality for patients with STEMI during the COVID-19 pandemic. In contrast, the in-hospital mortality of patients with NSTEMI did not significantly differ during the pandemic [31]. However, the influence of the COVID-19 pandemic on the prognosis of patients with AMI differs within the target population.

Our study has strength in its comprehensive presentation of detailed information, encompassing patient characteristics, treatment information, and laboratory data obtained through a thorough review of all medical records of patients with AMI. This study estimated the 2.5-year survival proportion of patients with AMI during both the pre-pandemic and pandemic periods. While numerous studies have investigated crude in-hospital or short-term mortality, only a few studies have investigated the cumulative survival proportion over a long period and employed a multivariate regression model for analysis [16-18].

However, this study has three limitations. First, the relatively small sample size may limit the statistical power to detect differences in the prognosis of patients with AMI between the pre-pandemic and pandemic periods. A large sample size might provide a significantly worse outcome during the pandemic period compared with that during the pre-pandemic period. Second, we cannot completely exclude the risk of selection bias because the study population only comprised patients admitted to our hospital. This study did not include patients with AMI admitted to other hospitals within the same prefecture. This may limit the generalizability of the findings. Third, confounding factors could influence the outcomes. However, we performed multivariate regression analysis adjusting for prognostic factors. Multivariate regression analysis showed concordant results with univariate analysis.

## Conclusions

The long-term prognosis of patients with AMI during the COVID-19 pandemic remains unclear. In this study, we reported that the 2.5-year MACE-free survival and overall survival rates of the patients with AMI admitted during the COVID-19 pandemic were not significantly different from those during the pre-pandemic period. The impact of the COVID-19 pandemic on the prognosis of patients with AMI appears to vary according to the study population.

Future research investigating the prognosis of patients with AMI admitted after this study period would be useful for understanding the longitudinal impact of the COVID-19 pandemic. The findings of this study will contribute to the planning of healthcare strategies for managing AMI during pandemics caused by unknown pathogens in the future.
